# Nutritional Stress Induced by Amino Acid Starvation Results in Changes for Slc38 Transporters in Immortalized Hypothalamic Neuronal Cells and Primary Cortex Cells

**DOI:** 10.3389/fmolb.2018.00045

**Published:** 2018-05-08

**Authors:** Sofie V. Hellsten, Rekha Tripathi, Mikaela M. Ceder, Robert Fredriksson

**Affiliations:** Molecular Neuropharmacology, Department of Pharmaceutical Biosciences, Uppsala University, Uppsala, Sweden

**Keywords:** SLC38 transporters, amino acid starvation, gene expression, protein expression, glutamine transporters

## Abstract

Amino acid sensing and signaling is vital for cells, and both gene expression and protein levels of amino acid transporters are regulated in response to amino acid availability. Here, the aim was to study the regulation of all members of the SLC38 amino acid transporter family, *Slc38a1-11*, in mouse brain cells following amino acid starvation. We reanalyzed microarray data for the immortalized hypothalamic cell line N25/2 subjected to complete amino acid starvation for 1, 2, 3, 5, or 16 h, focusing specifically on the SLC38 family. All 11 *Slc38* genes were expressed in the cell line, and *Slc38a1, Slc38a2*, and *Slc38a7* were significantly upregulated at 5 h and most strongly at 16 h. Here, protein level changes were measured for SLC38A7 and the orphan family member SLC38A11 which has not been studied under different amino acid starvation condition at protein level. At 5 h, no significant alteration on protein level for either SLC38A7 or SLC38A11 could be detected. In addition, primary embryonic cortex cells were deprived of nine amino acids, the most common amino acids transported by the SLC38 family members, for 3 h, 7 h or 12 h, and the gene expression was measured using qPCR. *Slc38a1, Slc38a2, Slc38a5, Slc38a6, Slc38a9*, and *Slc38a10* were upregulated, while *Slc38a3* and *Slc38a7* were downregulated. *Slc38a8* was upregulated at 5 h and downregulated at 12 h. In conclusion, several members from the SLC38 family are regulated depending on amino acid levels and are likely to be involved in amino acid sensing and signaling in brain.

## Introduction

Amino acid sensing and signaling is important for cells to control metabolism and protein synthesis, as well as catabolism and autophagy (Hyde et al., 2003; Kilberg et al., 2005). The amino acid sensing machinery is mainly mediated via the mechanistic target of rapamycin complex 1 (mTORC1) and the amino acid response (AAR) pathway (Gallinetti et al., 2013; Efeyan et al., 2015). These pathways are activated depending on amino acid availability and when the cells have plenty of amino acids, the mTORC1 pathway is activated to maintain protein synthesis and cellular growth (Laplante and Sabatini, 2012). The RAG kinase complex (Rag A/B and Rag C/D) are activated and translocate mTORC1 to the lysosomes where it binds the Ragulator complex. The pathway is fully activated when the lysosomal GDP bound protein Rheb becomes GTP bound in response to growth factors and encounters the mTORC1 complex (Jewell et al., 2013). Contrary, when the cells have scarce levels of amino acids the AAR pathway is activated, leading to reduced protein synthesis (Efeyan et al., 2015). The GCN2 kinases senses the limitation by binding to uncharged tRNAs (Deval et al., 2009) and phosphorylates the eukaryotic initiation factor 2α (eiF2α) which strongly inhibits the cap dependent translation (Harding et al., 2000; Zhang et al., 2002). The activating transcription factor 4 (ATF4) is upregulated by repressed translation (Averous et al., 2004) and bind elements termed amino acid response elements (AAREs) or nutrient sensing response elements (NSREs) in other genes. These elements are short nucleotide sequences and genes holding these are hence transcriptionally upregulated (Barbosa-Tessmann et al., 2000; Fafournoux et al., 2000; Bruhat et al., 2002).

Amino acids are important regulators of gene expression (Fafournoux et al., 2000) and numerous amino acid transporters are transcriptionally regulated in response to amino acid levels. These transporters are suggested to function as tranceptors, as they can both function as transporters as well as amino acid sensors (Hundal and Taylor, 2009). Several amino acid transporters from the Solute carrier (SLC) superfamily are transcriptionally altered depending on amino acid levels (Taylor, 2014). The SLCs are the largest group of transporters in human and comprises over 400 members divided into 65 families (Hediger et al., 2013)^1^. The members in each SLC family share at least 20 % protein sequence identity with another family member, and therefore also functional characteristics (Hediger et al., 2004). The SLCs are membrane-bound uniporters, symporters, or antiporters, with a diverse substrate profile and transport, among others, amino acids, sugars, fatty acids, vitamins, hormones, ions, and drugs (Hediger et al., 2013). One of the amino acid transporter families is the SLC38 family; the sodium coupled neutral amino acid transporter (SNAT) family, which holds 11 members, encoded by the genes *Slc38a1-11* (Sundberg et al., 2008). All members are functionally characterized (Bröer, 2014; Rebsamen et al., 2015; Hellsten et al., 2017b) except for SLC38A6 and SLC38A11, but SLC38A6 is histologically characterized in mouse brain (Bagchi et al., 2014). These transporters translocate small neutral amino acids, mostly glutamine, alanine, and asparagine (Bröer, 2014). Members from this family are shown to be involved in amino acid sensing and signaling, and SLC38A2 was upregulated on both gene and protein levels after amino acid starvation in BeWo cells (Novak et al., 2006). In the *Slc38a2* gene an AARE is identified in the first intron (Palii et al., 2006). Moreover, SLC38A9 is located to lysosomes and it is a component of the Ragulator-Rag complex responsible for amino acid sensing and activation of the mTORC1 (Jung et al., 2015; Rebsamen et al., 2015; Wang et al., 2015). SLC38A1 was recently found to be regulated in an amino acid responsive way (Bröer et al., 2016). In brain, several of the family members are proposed to participate in the glutamate/GABA-glutamine cycle which occurs between neurons and astrocytes (Bröer, 2014; Scalise et al., 2016).

In this study the aim was to study the transcriptional regulation of the SLC38 family members in a mouse neuronal cell-line following amino acid starvation, to identify which transporters could be involved in amino acid sensing and signaling in brain. Data from expression microarrays of the N25/2 cell-line exposed to complete amino acid starvation from a previous study (Hellsten et al., 2017c)was reanalyzed specifically for the SLC38 family. We also investigate regulatory changes at the protein level of SLC38A7 using western blot, since this member was transcriptionally upregulated in the hypothalamic cell line and has not previously been studied. In addition, changes at the protein level were also studied for SLC38A11using western blot which was chosen because this is the only family member that is still orphan. In addition, primary embryonic cortex cells were deprived of nine amino acids, glycine, L-alanine, L-asparagine, L-glutamine, L-histidine, L-isoleucine, L-leucine, L-serine, and L-valine, the most common amino acids transported by the SLC38 members and the gene expression changes were measured for the entire family using qPCR.

## Materials and methods

### Heat map analysis

Genesis version 1.7.6 was used to generate both heat maps. The microarray and analysis of data was performed in Hellsten et al. (2017c) and the array data can be accessed from the NCBI-GEO database with accession number GSE61402. The heat map displaying the gene expression changes measured with microarray for the *Slc38* genes in the immortalized hypothalamic cell line N25/2 was obtained by using the difference in log2 gene expression scores between the starved and control cells. *Slc38a1*, S*lc38a9*, and *Slc3810* had two probes each on the DNA chip and the expression score from both probes were used in the heat map analysis. The heat map presenting the gene expression changes measured with qPCR after partial amino acid starvation in primary cortex cells was generated by using the difference between the normalized mean values of expression in starved and control cells.

### Culturing and complete amino acid starvation of the immortalized hypothalamic cell line N25/2

The starvation experiment was performed in Hellsten et al. (2017c). Briefly, the mouse immortalized embryonic hypothalamic cell line N25/2, (mHypoE-N25/2, CEDARLANE, Canada) was cultured in Dulbecco's modified Eagles medium (DMEM) supplemented with 10% Fetal Bovine Serum (FBS), 1% Penicillin-Streptomycin and 1% Fungizone® Antimycotic at 37°C in 5% CO_2_, 95% air. Cells were grown in Nunclon surface dishes 150 × 20 mm (Thermo Scientific, USA) to 70–90% confluency before starvation experiment. Medium for the experiment was prepared using Earle's balanced salt solution (EBSS) supplemented with 1 mM Sodium Pyruvate 100 mM, 4X MEM Vitamin Solution. The control medium was as well supplemented with 0.4 mM glycine, 0.4 mM L-arginine, 0.2 mM L-cystine, 4.0 mM L-glutamine, 0.2 mM L-histidine, 0.8 mM L-isoleucine, 0.8 mM L-leucine, 0.8 mM L-lysine, 0.2 mM L-methionine, 0.4 mM L-phenylalanine, 0.4 mM L-serine, 0.8 mM L-threonine, 0.08 mM L-tryptophan, 0.4 mM L-tyrosine and 0.8 mM L-valine (Sigma-Aldrich, USA). The complete DMEM medium was replaced with starvation EBSS medium or control EBSS medium. The cells were incubated in the different media for 1, 2, 3, 5, or 16 h before RNA was extracted using RNeasy Midi Kit (Qiagen, Germany), following the manufacturers protocol. The samples from 1, 2, 3, and 16 h were run in singlets in each treatment group (starved and control), while the samples from 5 h were run in quadruplicates. Protein was extracted from three replicates in each treatment group from 5 h using the Allprep® DNA/RNA/Protein Mini kit (Qiagen, Germany) following manufacturers protocol. The protein concentration was measured using the Protein Quantification kit-Rapid (Sigma-Aldrich, USA) in FALCON® 96 well Clear Microtest Plate (Corning, USA) in FLUOstar Omega (BMG LABTECH, Germany) with the Omega MARS software.

### Western blot analysis of protein expression in the hypothalamic cell line N25/2

Ten microliters (~9–11 μg) of protein samples were diluted in 15 μl of sample buffer [95% 2 × Lammeli's sample buffer (Bio-Rad, USA), 5% 2-mercaptoethanol (Sigma-Aldrich, USA)] was added and the samples were incubated at 95°C for 5 min. Twenty-five microliters of samples were loaded in wells together with 15 μl Page ruler™ Prestained Protein ladder, 10–180 kDa (Thermo Fisher Scientific, USA). Electrophoresis was performed at 250 V for approximately 25 min with gel Mini-protean TGX Precast Gels 4–15%, 10 well comb, 50 μl/well (Bio-Rad, USA) with running buffer (0.025 M Trizma base, 0.192 M Glycine, 0.1% SDS). Proteins were transferred to a 0.2 μm PVDF membrane using the Trans-Blot® Turbo™ Mini PVDF Transfer Packs (Bio-Rad, USA) in the Trans-Blot® Turbo™ Transfer System for **7** min (Bio-Rad, USA). The membrane was incubated in blocking buffer [5% Blotting grade blocker Non-fat dry Milk (Bio-Rad, USA) in TTBS (0.15 M NaCl, 0.01 M Trizma base, 0.05% Tween-20, pH = 8.0)] for 1 h, before incubation in the custom made polyclonal anti-SLC38A7 (produced in rabbit) (Innovagen, Sweden) (NH_2_-CVMSKEPDGASGSPW-CONH2), which was used in Hägglund et al. (2011) diluted 1:200 or the custom made polyclonal anti-SLC38A11 (produced in rabbit) (Innovagen, Sweden) (MSYQQPQLSGPLQRC) diluted 1:100 with β-actin (produced in mouse) (Sigma-Aldrich, USA) diluted 1:1000 in blocking buffer overnight at 4°C. The membrane was washed 3 × 10 min in TTBS before 1 h incubation in goat-anti-rabbit horseradish peroxidase antibody (Invitrogen, USA) diluted 1:5000 in blocking buffer. The membrane was washed 4 × 10 min in TTBS and the blot was developed using Clarity Western ECL Substrate and visualized using a CCD camera (Bio-Rad, USA). For SLC38A7 the membrane was washed 6 × 10min in TTBS after development and the membrane was incubated in β-actin diluted 1:1000 in blocking buffer overnight and the membrane was treated as stated above. With the exception that the membrane was incubated in goat-anti-mouse horseradish peroxidase antibody (Invitrogen, USA) diluted 1:10000 for 1 h. For SLC38A11 the membrane was washed in TTBS 6 × 10 min after development and incubated in goat-anti-mouse horseradish peroxidase antibody (Invitrogen, USA) diluted 1:10000 in blocking buffer followed by development as stated above.

### Calculations of normalized protein expression

The western blots were quantified using ImageJ software and the protein expression was normalized against β-actin. GraphPad Prism 5 (Graph Pad software, USA) was used to generate graphs and for statistical calculations. Unpaired *t*-test were performed with significance levels (^*^≤0.05, ^**^≤0.01, ^***^≤0.001).

### Ethical statement

Experiments including mice were approved by local ethical committee in Uppsala (Uppsala Djurförsöksetiska Nämnd, Uppsala district court) (Permit Number C67/13), following the guidelines of European Communities Council Directive (2010/63). C57Bl6/J mice (Taconic M&B, Denmark) were used and the mice had free access to water and standard R3 chow (Lantmännen, Sweden). The mice were housed in a temperature, light/dark, and humidity controlled room. Mice were mated in the animal facility and pregnancies were confirmed by the mucus plug.

### Preparation and partial amino acid starvation of primary embryonic cortex cells

The preparation and starvation of primary cells were performed as described in Perland et al. (2017). Briefly, a pregnant female mouse was euthanized by cervical dislocation at embryonic day e14.5. Cortices were dissected from the embryos and placed in ice cold PBS (137.0 mM NaCl, 2.7 mM KCl, 8.1 mM Na_2_HPO_4_) supplemented with 10.0 mM glucose (all from Sigma-Aldrich, USA). The tissues were chemically dissociated using a DNase/Papain solution prior mechanical dissociation. Single cells were filtered through a 70 μm nylon cell strainer (BD Stockholm, Sweden) before plated at a density of 7.5 × 10^4^-1.5 × 10^5^ cells per well in PLL coated 6 well plates (Invitrogen, USA) using plating media. Following cell adhering, the plating media was replaced with growth media supplemented with B27 (Gibco®, Life technologies, USA). 75% of the medium was changed every third day and the cells grew for 10 days before starvation experiment. The control medium was prepared using EBSS (Gibco®, Life technologies, USA) supplemented with 2.0 mM GlutaMAX™ and the amino acids glycine, L-alanine, L-arginine, L-asparagine, L-cysteine, L-histidine, L-isoleucine, L-leucine, L-lysine, L-methionine, L-phenylalanine, L-proline, L-serine, L-threonine, L-tryptophan, L-tyrosine, and L-valine (Sigma-Aldrich, USA) was added in the same concentrations as in the Neurobasal® A medium. The starved medium was prepared using EBSS supplemented with L-arginine, L-cysteine, L-lysine, L-methionine, L-phenylalanine, L-proline, L-threonine, L-tryptophan, and L-tyrosine (Sigma-Aldrich, USA) in the same concentrations as in the Neurobasal® A medium. Both the control and starvation medium was supplemented with 1.0 mM Sodium-Pyruvate, 1% Penicillin-Streptomycin, 2% B-27® (50X), 4X MEM Vitamin Solution (100X) (Gibco®, Life technologies, USA) and 10.9 mM HEPES (1 M) buffer solution (Gibco®, Life technologies, USA). Hence, the starved cells were deprived of glycine, L-alanine, L-asparagine, L-glutamine, L-histidine, L-isoleucine, L-leucine, L-serine and L-valine, which are among the most common amino acids transported by the SLC38 family and their precursors. The experiment was run in triplicates in each treatment group (starved vs. control cells) and the cells were treated in the limited amino acid medium or the complete amino acid medium for 3, 7, or 12 h before RNA was extracted using RNeasy Midi Kit (Qiagen, Germany), following the manufacturers protocol. cDNA synthesis was performed using the High-Capacity RNA-to-cDNA kit (Invitrogen, USA) according to the manufacturers protocol and the cDNA from the triplicates in each treatment group were pooled. The cDNA concentrations were measured using a ND-spectrophotometer (NanoDrop Technologies, USA).

### qPCR analysis of gene expression in primary embryonic cortex cells

The cDNA samples were analyzed using qPCR on MyiQ thermal cycler (Bio-Rad Laboratories, Sweden). Primers were designed using Beacon Designer v.8 (Premier Biosoft, USA) and the primers used are listed in Table 1. Housekeeping genes used for normalization were mouse *m*β*-Actin, mGlycerylaldehyde 3-phosphate dehydrogenase* and *mHistone 3a*. Sixty nanograms cDNA per qPCR reaction was combined with 0.05 μl of each primer (100 pmol/μl), 3.6 μl 10X DreamTaq buffer (Thermo Fischer Scientific), 0.2 μl of 25 mM dNTP mix (Thermo Fischer Scientific), 1μl DMSO, 0.5 μl SYBR Green (Invitrogen) and 0.08 μl of Dream Taq (5U/μl, Thermo Fisher scientific). The volume was adjusted to 20 μl with water. The amplification was performed as follow; initial denaturation, 95.0°C for 30s, 45 cycles of: 95.0°C for 10s, 55.8–60.0°C for 30s and 72.0°C for 30s. Cycling was followed by melt curve performance for 81 cycles, starting at 55.0°C, with steps of 0.5°C and 10s intervals. All qPCRs were run in triplicates and water was used as a negative control.

**Table 1:** Primers used for qPCR.

**Primer**	**Forward/Reverse**
*Slc38a1*	F:tga cga gag tca cgc aga gat g, R:gag cag tat gag aac aag cga agc
*Slc38a2*	F:tct act cgc tgg ttc ttc, R:aat aaa ctt gtc act tcc ctt
*Slc38a3*	F:act ctt gtc ttc ttc cct ctc ctc, R:gcc tcc ctt ctc cca gca g
*Slc38a4*	F:tct cac tct aca cca aca cta agg, R:act cta tac tgg caa ccg tca ttc
*Slc38a5*	F:tgg agg tgt ctg gtc tct aat aa, R:ggc agt gag gca act cta agg
*Slc38a6*	F:gga aga aca cca cag acc aga atc, R:tgc tct ctt gcc tct tgc tct c
*Slc38a7*	F:att gtt gtt ctc cca tcc atc cc, R:act gtg aaa ggc agc act tgg
*Slc38a8*	F:cgt ggt gac tcg gga cag, R:ta caa gcc agg gac act aag g
*Slc38a9*	F:ttg aaa gcg agg gaa atg atg gtc, R:atg gga atg agg gtc act gag aag
*Slc38a10*	F:tgg tga agg ctc cga aga aag g, R:act tgg ctt ggg tct gaa ctg g
*Slc38a11*	F:act ttc aat tcg gaa cct, R:cat cag tgc taa tct tgt g
*mβ-Actin (mActb)*	F:cct tct tgg gta tgg aat cct gtg, R:cag cac tgt gtt ggc ata gag g
*mGlycerylaldehyde 3-phosphate dehydrogenase (mGAPDH)*	F:gcc ttc cgt gtt cct acc, R:gcc tgc ttc acc acc ttc
*mHistone3a (mH3a)*	F:cct tgt ggg tct gtt tga, R:cag ttg gat gtc ctt ggg

### Data analysis and normalized expression calculations

The MyIQ software (Bio-Rad Laboratories, Sweden) was used to obtain the qPCR cycle threshold (Ct) -values and melt curve data. The melting curves were compared to the negative control to verify that only one product was amplified. The triplicates for the raw Ct-values were compared and excluded if the difference was greater than 0.5. For *Slc38a4* and *Slc38a11*, no Ct outliers could be removed due to high Ct values (Ct > 35). The efficiency for each primer pair was determined using LinRegPCR v7.5 and the average qPCR primer efficiency and standard deviation were calculated after significant outliers were removed using Grubbs outlier test (GraphPad Software, USA). The delta Ct-method was used to transform the Ct-values into relative quantities with standard deviations for each treatment time. Geometric means of all housekeeping genes were calculated and the normalized mRNA levels were calculated by dividing the relative Ct-values of the sample by the geometric mean of the housekeeping genes relative Ct-values. Unpaired *t*-tests (^*^≤0.05, ^**^≤0.01, ^***^≤0.001) were performed using GraphPad Prism 5 (GraphPad Software, USA) between the control cells and the starved cells.

## Results

### Microarray analysis of gene expression in the immortalized hypothalamic cell line N25/2

**Figure 1:**
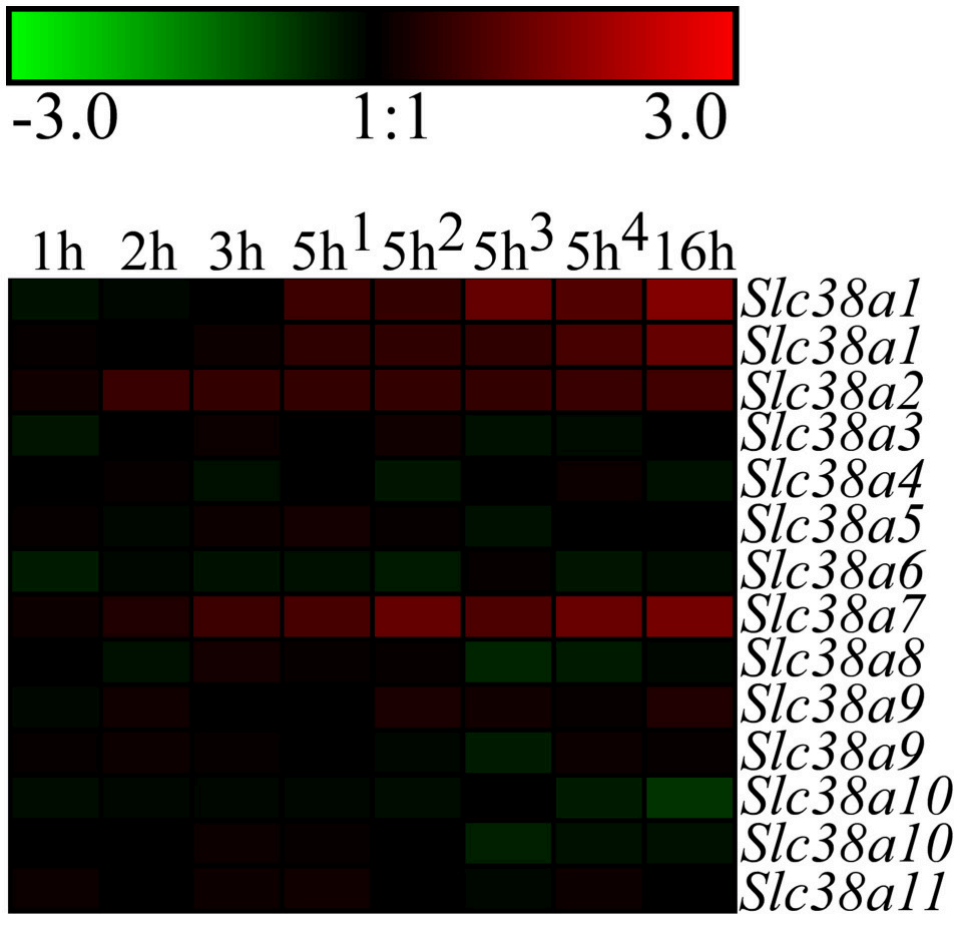
Heat map analysis of the gene expression changes for *Slc38a1-11* after complete amino acid starvation performed on the immortalized hypothalamic cell line N25/2. A heat map of the gene expression changes measured with microarray between starved cells and controls at 1, 2, 3, 5, or 16 h. The color scale represents the log2 difference between starved and control cells. Green color represents downregulation and red color represent upregulation of gene expression. Note that *Slc38a1, Slc38a9*, and *Slc3810* had two probes each on the gene chip and the expression scores from both probes are presented in the heat map.

The immortalized hypothalamic cell line N25/2 was completely starved of amino acids and the gene expression was analyzed using microarray in Hellsten et al. (2017c). All 11 genes encoding members of the SLC38 family were expressed in the cell line (expression score > 5.0). *Slc38a1*, S*lc38a9*, and *Slc38a10* had two probes each on the microarray and the results were comparable between the probes. The regulation of the *Slc38* genes is presented in the heat map (Figure 1). The red color represents upregulation and green color represents downregulation of gene expression. In Table 2, all differences in log2 expression scores and the adjusted *P*-value at 5 h of starvation, and in addition the system, substrates and location in brain for all family members are presented. *Slc38a1, Slc38a2*, and *Slc38a7* were significantly upregulated at 5 h (i.e., adj. *P*-value < 0.01) of amino acid starvation, and strongly upregulated at 16 h of starvation. *Slc38a3, Slc38a4, Slc38a5, Slc38a6, Slc38a8, Slc38a9, Slc38a10*, and *Slc38a11* were not significantly changed at 5 h in the array.

**Table 2:** Summary of the microarray data and the SLC38 transporters.

**Gene**	**1 h**	**2 h**	**3 h**	**5 h^1^**	**5 h^2^**	**5 h^3^**	**5 h^4^**	**Adj. *P*-value (5 h)**	**16 h**	**System A/N**	**Substrates**	**Main location in brain**
*Slc38a1*	−0.25	−0.08	−0.02	0.72	0.64	1.17	1.00	0.00009461	1.52	A	Gln, Ala, Asn, Cys, His, Ser	GABAergic neurons (Solbu et al., 2010)
	0.07	0.02	0.14	0.55	0.59	0.56	0.83	0.00010526	1.22			
*Slc38a2*	0.23	0.68	0.63	0.64	0.64	0.63	0.68	0.00001176	0.78	A	Ala, Asn, Cys, Gln, Gly, His, Met, Pro, Ser	Glutamatergic neurons (González-González et al., 2005)
*Slc38a3*	−0.28	−0.02	0.014	0.06	0.20	−0.20	−0.13	0.93754260	−0.01	N	Gln, His, Ala, Asn	Astrocytes (Boulland et al., 2003)
*Slc38a4*	−0.02	0.07	−0.20	−0.07	−0.29	−0.05	0.11	0.73438555	−0.21	A	Ala, Asn, Cys, Gly, Ser, Thr	?
*Slc38a5*	0.07	−0.13	0.15	0.26	0.06	−0.23	−0.04	0.96736096	−0.04	N	Gln, Asn, His, Ser	Astrocytes (Cubelos et al., 2005)
*Slc38a6*	−0.36	−0.10	−0.24	−0.21	−0.32	0.06	−0.29	0.12927331	−0.15	?	?	Glutamatergic neurons (Bagchi et al., 2014)
*Slc38a7*	0.17	0.34	0.72	0.86	1.17	0.91	1.25	0.00002241	1.38	N (Hägglund et al., 2011)	Gln, His, Ser, Ala, Asn	GABAergic and glutamatergic neurons (Hägglund et al., 2011)
*Slc38a8*	0.00	−0.21	0.26	0.10	0.08	−0.45	−0.31	0.49605581	−0.10	A (Hägglund et al., 2015)	Gln, Arg, Ala, Asp, Leu, His, Asn, Pro, Glu	GABAergic and glutamatergic neurons (Hägglund et al., 2015)
*Slc38a9*	−0.13	0.18	0.01	−0.01	0.33	0.22	0.09	0.26471418	0.37	?	Arg, Gln, His, Pro, Lys, Glu, Leu	GABAergic and glutamatergic neurons (Hellsten et al., 2017a)
	0.07	0.17	0.09	0.03	−0.09	−0.36	0.17	0.68362400	0.10	?		
*Slc38a10*	−0.15	−0.09	−0.08	−0.08	−0.17	−0.06	−0.36	0.16057397	−0.62	A (Hellsten et al., 2017b)	Gln, Glu, Ala, D-Asp, Ser (Hellsten et al., 2017b)	Neurons and astrocytes (Hellsten et al., 2017b)
	0.02	0.02	0.12	0.09	−0.04	−0.38	−0.22	0.30033341	−0.21			
*Slc38a11*	0.21	0.04	0.18	0.18	0.04	−0.10	0.17	0.65258319	−0.07	?	?	?

*Difference in log2 expression scores between the starved and control cells from the microarray analysis is presented (1–16 h), as well as the system, substrates and astrocytic/GABAergic/glutamatergic-neuronal location in brain for the transporters. Information about system classification and substrate profile were obtained from SLC tables (http://www.bioparadigms.org/slc/intro.htm) otherwise stated, (? Not known)*.

### Protein expression of SLC38A7 and SLC38A11 in the immortalized hypothalamic cell line N25/2

**Figure 2:**
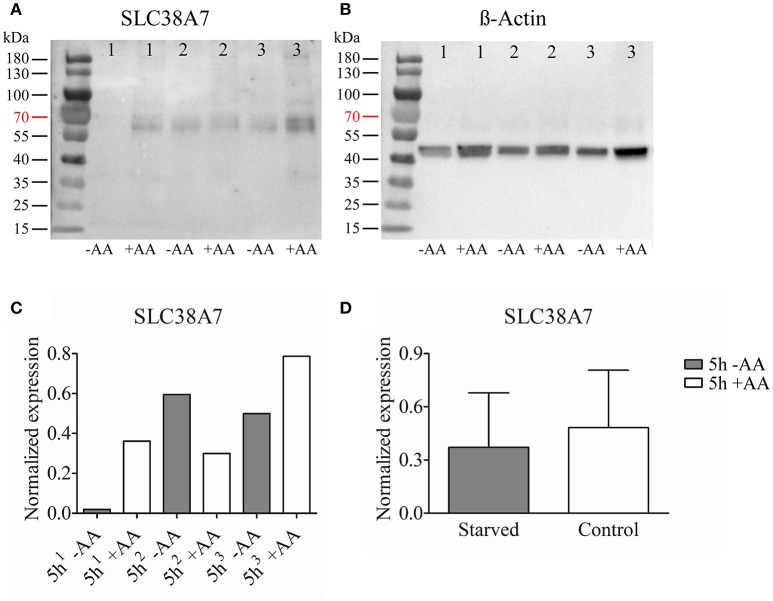
Western blot analysis of SLC38A7 protein expression in the immortalized hypothalamic cell line N25/2 after complete starvation at 5 h. **(A)** The blot (developed for 18.4 s) displays the protein expression of SLC38A7 in three replicates (1, 2, 3) from each treatment group. The predicted size of the mouse SLC38A7 protein is 49.9 kDA (463 amino acids, NP_766346) and a band with approximately size of 62 kDa was detected. **(B)** The blot (developed for 3.1 s) displays the protein expression of β-actin in each sample which was used for normalization of protein expression in each sample. **(C)** The graph illustrates the normalized protein expression of SLC38A7 in the starved cells compared with the amino acid treated controls for each replicate. **(D)** The graph represents the normalized protein expression of all replicates in each group (mean value of protein expression ± SD). An unpaired *t*-test was performed between the protein expression in starved cells and controls. No difference (*p* = 0.6886) was detected on protein level for SLC38A7.

**Figure 3:**
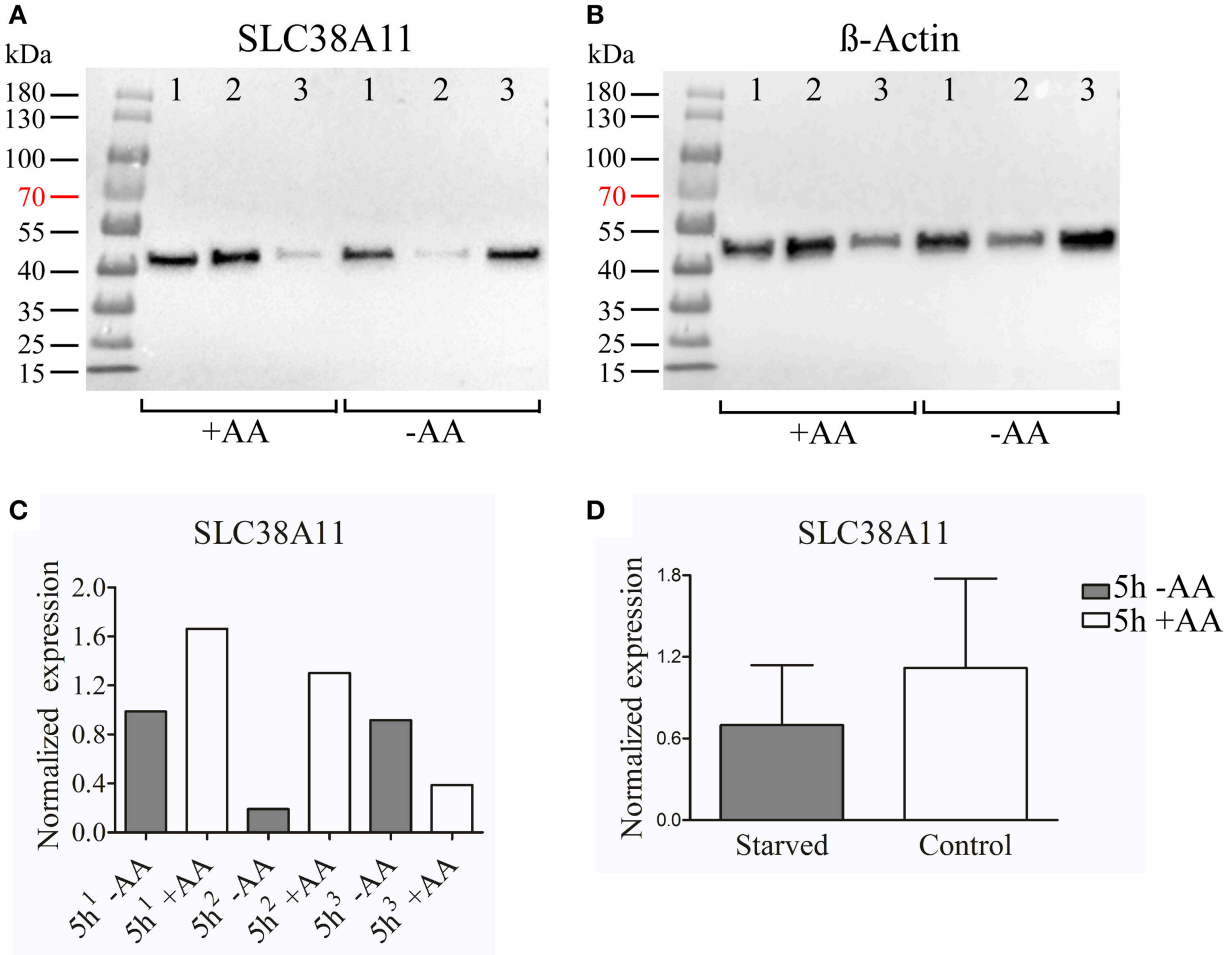
Western blot analysis of SLC38A11 protein expression in the immortalized hypothalamic cell line N25/2 after complete starvation at 5 h. **(A)** The blot (developed for 2.0 s) show the protein expression of SLC38A11 in three replicates (1, 2, 3) in each treatment group. The predicted size of the mouse SLC38A11 protein is 49.6 kDA (453 amino acids, NP_796048) and a band with approximate size of 46 kDA was detected. **(B)** The blot (developed for 2.0 s) visualizes the protein expression of β-actin which was used to normalize the protein expression in each sample. **(C)** The graph displays the normalized protein expression of SLC38A11 in the starved cells compared with the amino acid treated controls for each sample. **(D)** The graph represents the normalized protein expression of all replicates in each treatment group (mean value of protein expression ± SD). An unpaired *t*-test was performed between the protein expression in starved cells and controls. No difference (*p* = 0.4108) of protein expression for SLC38A11 was measured.

We chose to study SLC38A7 and SLC38A11 on protein level. This, because SLC38A7 was transcriptionally upregulated in the hypothalamic cell line and this transporter has not previously been studied on protein level. While the other two SLC38 family members (*Slc38a1* and *Slc38a2*) found to be transcriptionally upregulated in the N25/2 hypothalamic cells have previously been studied in several studies and therefore we found it most interesting to investigate the regulation of SLC38A7. In addition, SLC38A11 is currently the only family member that has not been either studied histologically or functionally so therefore we found it interested to study this transporter on protein level in the hypothalamic cells. The protein expression of SLC38A7 was analyzed using western blot and a band was detected with approximate size of 62 kDa in all samples except first replicate of starved cells (Figure 2A). β-actin was used for normalization and the blot is displayed in Figure 2B. SLC38A7 was upregulated in the second sample while downregulated in the third sample of starved cells compared with the controls. In the first starved sample, SLC38A7 was not detected, however β-actin was detected. The normalized protein levels for SLC38A7 were calculated in each replicate (1, 2 and 3) in each treatment group (Figure 2C). The expression of SLC38A7 was combined in the starved cells and control cells (mean value normalized protein expression ± SD) and overall the protein expression of SLC38A7 was not significantly changed (*p* = 0.6886; Figure 2D). The protein expression of the orphan family member SLC38A11 was analyzed using western blot and in the blot a band with approximate size of 46 kDa was detected (Figure 3A). The predicted size of SLC38A11 in mouse is 49.6 kDA (453 amino acids, NP_796048) and hence the blot indicates specific binding of the anti-SLC38A11 antibody. β-actin was used to normalize the protein expression and was detected in all replicates in each treatment group (Figure 3B). SLC38A11 was downregulated after starvation in the first and second sample, while upregulated in the third sample (Figure 3C). The expression of SLC38A11 was combined in the starved cells and control cells (mean value normalized protein expression ± SD) and these results indicate that the protein expression of SLC38A11 was unchanged (*p* = 0.4108; Figure 3D).

### qPCR analysis of gene expression in the primary embryonic cortex cells

**Figure 4:**
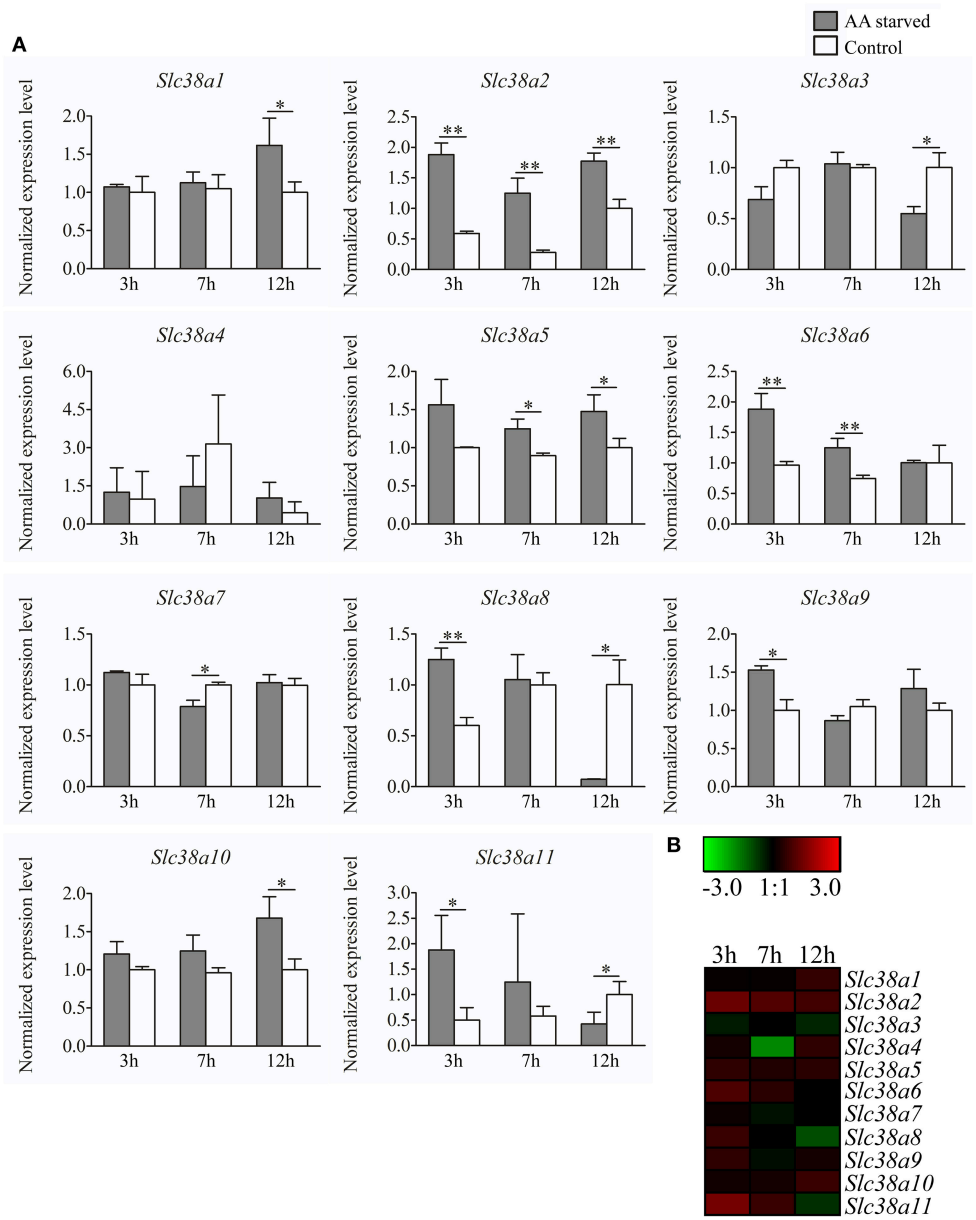
Gene expression analysis of *Slc38a1-11* in primary cortex cells at 3h, 7h and 12h treatment. **(A)** Gene expression data from the primary cortex cells using qPCR for *Slc38a1-11*. The gene expression levels were normalized against *m*β*-Actin, mGlycerylaldehyde 3-phosphate dehydrogenase*, and *mHistone 3a* and the normalized expression level ± SD (*n* = 3) is presented for each gene. Dark gray bars represent amino acid starved cells and white bars represent amino acid treated controls. Unpaired t-tests were used between starved cells and controls (^*^≤0.05, ^**^≤0.01) to analyze alteration in gene expression. The x-axis represents time of treatment in hours and the y-axis represents the normalized mRNA expression levels. **(B)** Heat map analysis summarizing the gene expression alterations measured with qPCR for *Slc38a1-11*. The color scale represents the difference between the mean value of normalized expression in starved and control cells. Green color represents downregulation and red color represent upregulation of gene expression.

Primary cortex cells were partly deprived of the most common amino acids transported by the SLC38 family, as well as their precursors, for 3, 7, and 12 h. Subsequently, the gene expression was measured with qPCR for *Slc38a1-11* (Figure 4A). All genes were detected in the primary cells, however *Slc38a4* and *Slc38a11* had low mRNA expression (i.e., CT>35) and these results should hence be considered with caution. *Slc38a1* was upregulated at 12 h (*p* = 0.0495). *Slc38a2* expression was increased at all three deprivation timepoints 3 h (*p* = 0.0012), 7 h (*p* = 0.0026), and 12 h (*p* = 0.0026). *Slc38a3* was downregulated at 12 h (*p* = 0.0158), *Slc38a4* expression was unchanged at all three timepoints and *Slc38a5* expression was increased at 7 h (*p* = 0.0159) and 12 h (*p* = 0.0304). *Slc38a6* expression was upregulated at 3 h (*p* = 0.0079) and 7 h (*p* = 0.0057). *Slc38a7* expression was decreased at 7 h (*p* = 0.0054) and*Slc38a8* was upregulated at 3 h (*p* = 0.0059) of deprivation but downregulated at 12 h (*p* = 0.0323). *Slc38a9* was upregulated early at 3 h (*p* = 0.0165) while *Slc38a10* was upregulated late at 12 h (*p* = 0.0201). *Slc38a11* expression was increased at 3 h (*p* = 0.0295) and decreased at 12 h (*p* = 0.0455). The heat map (Figure 4B) summarizes the gene expression changes measured with qPCR for each *Slc38* gene.

## Discussion

Amino acid sensing and signaling are crucial for cells to control basic functions and transporters involved in these processes often act as amino acid sensors (Efeyan et al., 2015). In many cancer cells, amino acid transporters are upregulated to enable rapid cell growth and are therefore potential drug targets e.g. as SLC1A5, SLC6A15, SLC7A5, and SLC7A11 (Bhutia et al., 2015). Here, complete and partial amino acid starvation of cells was performed to study the regulation of the amino acid transporters in the SLC38 family in mouse brain cells. The hypothalamic cell line N25/2 was used to measure changes in expression levels on gene and protein levels during complete amino acid starvation, and primary cortex cells, comprising both neurons and astrocytes, were partly deprived of amino acids to study gene expression changes. A murine hypothalamic cell line and primary cells from different brain regions were used in this study to obtain a more comprehensive understanding of the regulation. *Slc38a2* was upregulated in the hypothalamic cell line and at all three timepoints in the primary cortex cells. *Slc38a2* is known to be transcriptionally induced by amino acid starvation (Gaccioli et al., 2006) and this gene holds an AARE (Palii et al., 2006). SLC38A2 follows adaptive regulation in response to amino acid stress by GCN pathway (Taylor, 2014). Along with this *Slc38a9* is also known as an arginine sensor and regulate the mTORC1 pathway. Moreover, *Slc38a1* was also transcriptionally induced in both cell types, however only late in the primary cortex cells, and SLC38A1 expression was recently found to be regulated by amino acid starvation (Bröer et al., 2016). SLC38A1 and SLC38A2 are mostly expressed on neurons in brain and the main function of these proteins are suggested to be uptake of glutamine in GABAergic respectively glutamatergic neurons (Bak et al., 2006). In cancer cells, SLC38A1 and SLC38A2 are involved in providing glutamine for glutaminolysis (Bröer et al., 2016). Both *Slc38a1* (Mackenzie et al., 2003) and *Slc38a2* (Yao et al., 2000) encode system A transporters, which system *Slc38a8* (Hägglund et al., 2015) also is classified to. *Slc38a8* was also initially upregulated in the primary cortex cells. Among transporters classified to system N, *Slc38a7* (Hägglund et al., 2011) showed increased expression in the hypothalamic cell line while reduced in the cortex cells. The dissimilarity in regulation pattern could be due to different starvation times and the fact that the hypothalamic cells are completely starved while the cortex cells are partly deprived of amino acids, also the cultures are inherently different, because the primary cultures are embryonic and mixed cultures of neurons and glia cells, while the N25/2 cultures are only neurons. The gene expression of the functionally orphan protein SLC38A6 was upregulated in the primary cortex cells. In a previous study, this transporter was shown to be expressed specifically on glutamatergic neurons (Bagchi et al., 2014). Assuming SLC38A6 is a transporter for glutamine as speculated in Bagchi et al. (2014), its role could be in importing glutamine into the presynapes of glutamatergic neurons, where it will be used as substrate for glutamine synthesis. If so, it makes sense that SLC38A6 is upregulated in response to lack of glutamine in the starved state, so neurons can maintain glutamine levels in the pre-synapse. SLC38A7 (Hägglund et al., 2011) and SLC38A8 (Hägglund et al., 2015) are suggested to facilitate uptake of glutamine in neurons and the expression levels of these transcripts were altered in the cortex cells. An interesting point of view is that it is possible that *Slc38* genes encodes proteins with similar localization and function, are regulated differently; as one protein could have housekeeping functions while the other protein is responding to stress. For example, SLC38A3 and SLC38A5 have similar expression and function in brain, and these genes had altered gene expression in opposite direction. These proteins are also closely phylogenetically related (Hägglund et al., 2015). This theory can also be applied to SLC38A7 and SLC38A8 which also are phylogenetically closely related and co-expressed on most neurons (Hägglund et al., 2015) and initially regulated in the opposite direction after partial amino acid starvation, and in the hypothalamic cell line *Slc38a7* was transcriptionally induced while *Slc38a8* was not. In a previous study, SLC38A7 co-localized with lysosomes in HeLa cells and was found to mediate flux of glutamine and asparagine and hence crucial for growth of cancer cells (Verdon et al., 2017). SLC38A9 is located on lysosomes and is a component of the amino acid sensing Ragulator-Rag complex responsible for activation of mTORC1 (Jung et al., 2015; Rebsamen et al., 2015; Wang et al., 2015) and in mouse brain SLC38A9 immunostaining is detected in both GABAergic and glutamatergic neurons (Hellsten et al., 2017a). Expression of *Slc38a9* was not altered by complete amino acid starvation in hypothalamic cells but initially upregulated in the primary cortex cells. In a previous study where mice were food deprived for 24 h before euthanasia, *Slc38a9* was upregulated in cortex while unaffected in hypothalamus in brain (Hellsten et al., 2017a). A hallmark of the SLC38 family is adaptive regulation with modulator of nutrient stress, and these transporters are translocated within the cell, e.g., to and from the plasma membrane depending on different stimulus such as amino acid levels (Bröer and Gether, 2012; Bröer, 2014). Studying gene expression levels is a good approach to pinpoint which genes are responding to alterations in amino acid levels, but to understand how the regulation of gene expression affects the cellular function, alterations on protein levels are crucial to study. The system A transporters SLC38A1 (Bröer et al., 2016) and SLC38A2 (Novak et al., 2006) are known to be upregulated on protein level in response to amino acid starvation. Apart from SLC38A1 and SLC38A2 the only other transporter that had significantly changed expression at 5 h was *Slc38a7*. We investigated if these changes could also be detected on protein level and found that the protein expression of SLC38A7 was unchanged at 5 h in the hypothalamic cell line. We have however verified that *Slc38a7* was upregulated in the hypothalamic cell line at 5 h at the mRNA level using qPCR in Hellsten et al. (2017c). This is likely a result of the fact that changes in protein levels occurs later than on mRNA level and has not yet manifested itself at the timepoint we are measuring changes at the protein level. We also investigated changes in protein levels of SLC38A11, the last orphan member of the SLC38 family. The gene expression of *Slc38a11* was measured in a previous study and was overall low in the rat brain (Sundberg et al., 2008), and both in the hypothalamic cell line and the cortex cells the mRNA levels were low. However, SLC38A11 protein levels could easily be detected in the hypothalamic cell line, but no effect on mRNA and protein levels were detected.

Neurons have low ability for *de-novo* synthesis of glutamine and other amino acids compared to other cells and is therefore dependent on import of these compounds provided either by astrocytes or glia cells or from import into the brain via the blood brain barrier (Bixel et al., 2001; Lieth et al., 2001; Schousboe, 2018). Therefore, plasma membrane expressed transporters on neuronal cells are crucial for maintaining homeostasis of amino acids and this transport needs to be tightly controlled. The main way, as far as currently understood, of regulating transport mediated by SLCs is for the cells to change number of transporters by, in the short term, alter surface expression and over longer timespans regulate number of transporters at the transcription and protein synthesis levels. Nerve cells have five or possibly six transporters from the SLC38 family which could possibly contribute in importing glutamine into neurons (Schiöth et al., 2013; Bröer, 2014). The data presented in this paper shows that in a neuronal cell-line, SLC38A1, SLC38A2, and SLC38A7 are regulated in response to amino acid starvation. In the nervous system, SLC38A1 and SLC38A2 are found on certain neuronal populations while SLC38A7, together with SLC38A8, are ubiquitously expressed on most neurons (Hägglund et al., 2011). This suggests that glutamine import into neurons by the SLC38 family could be dependent on SLC38A8, which is ubiquitously expressed on neurons, as housekeeping transporter and SLC38A7 together with one of or both SLC38A1 and SLC38A2 for the regulated transport. Expression of SLC38A6 is not regulated in the N25/2 cells and this transporter is found only on excitatory neurons (Bagchi et al., 2014) suggesting a role specifically for importing glutamine for the glutamine/glutamate/GABA cycle. However, our data from primary cultures, which are mixed cultures of different neuronal subtypes and non-neuronal cells such as astrocytes and glia cells, which is closer to a true representation of the *in vivo* situation, suggests that the situation is much more complex *in vivo* because here we show that all SLC38 members except for Slc38a4 can be regulated in response to amino acid starvation. Interestingly, some are being upregulated and others downregulated in response to amino acid starvation. In the *in vivo* situation, the SLC38 family of transporters is most likely regulated differently on different cell-types to maintain glutamine homeostasis as close as possible for the brain in total.

## Conclusion

In conclusion, three (*Slc38a1, Slc38a2*, and *Slc38a7*) of eleven members from the SLC38 family, have increased transcription following 5 h of complete amino acid starvation in the hypothalamic cell line N25/2. However, no regulation of SLC38A7 could be measured on protein level at 5 h. Following partial amino acid starvation of primary embryonic mouse cortex cells genes encoding all 11 SLC38 family members except *Slc38a4* were altered. Genes encoding both system A and N transporters were transcriptionally regulated upon changes in amino acid levels. Several of the SLC38 family members are possibly involved in amino acid sensing and signaling in brain.

## Author contributions

SH wrote and drafted manuscript, planned and performed starvation experiments, microarray data analysis, generation of heat maps, qPCR and qPCR analysis, statistics, protein sample preparations and western blot; RT protein sample preparations, western blot, analysis of western blots and revised the draft paper; MC cDNA synthesis, qPCR and qPCR analysis; RF drafted manuscript, planned experiments. All authors have read and approved of the manuscript and helped with interpretation of results.

### Conflict of interest statement

The authors declare that the research was conducted in the absence of any commercial or financial relationships that could be construed as a potential conflict of interest.

## Footnotes

^1^http://slc.bioparadigms.org/
